# Identification and Characterization of the V(D)J Recombination Activating Gene 1 in Long-Term Memory of Context Fear Conditioning

**DOI:** 10.1155/2016/1752176

**Published:** 2015-12-30

**Authors:** Edgardo Castro-Pérez, Emilio Soto-Soto, Marizabeth Pérez-Carambot, Dawling Dionisio-Santos, Kristian Saied-Santiago, Humberto G. Ortiz-Zuazaga, Sandra Peña de Ortiz

**Affiliations:** ^1^Molecular and Cellular Cognition Laboratory, University of Puerto Rico, Rio Piedras Campus, San Juan, PR 00931-3360, USA; ^2^Functional Genomics Research Center, Department of Biology, University of Puerto Rico, Rio Piedras Campus, San Juan, PR 00931-3360, USA; ^3^Department of Computer Science, University of Puerto Rico, Rio Piedras Campus, San Juan, PR 00931-3360, USA; ^4^High Performance Computing Facility, Central Administration, San Juan, PR 00931, USA

## Abstract

An increasing body of evidence suggests that mechanisms related to the introduction and repair of DNA double strand breaks (DSBs) may be associated with long-term memory (LTM) processes. Previous studies from our group suggested that factors known to function in DNA recombination/repair machineries, such as DNA ligases, polymerases, and DNA endonucleases, play a role in LTM. Here we report data using C57BL/6 mice showing that the *V(D)J recombination-activating gene 1* (*RAG1*), which encodes a factor that introduces DSBs in immunoglobulin and T-cell receptor genes, is induced in the amygdala, but not in the hippocampus, after context fear conditioning. Amygdalar induction of *RAG1* mRNA, measured by real-time PCR, was not observed in context-only or shock-only controls, suggesting that the context fear conditioning response is related to associative learning processes. Furthermore, double immunofluorescence studies demonstrated the neuronal localization of RAG1 protein in amygdalar sections prepared after perfusion and fixation. In functional studies, intra-amygdalar injections of *RAG1* gapmer antisense oligonucleotides, given 1 h prior to conditioning, resulted in amygdalar knockdown of *RAG1* mRNA and a significant impairment in LTM, tested 24 h after training. Overall, these findings suggest that the *V(D)J recombination-activating gene 1*, *RAG1*, may play a role in LTM consolidation.

## 1. Introduction

Studies suggest that LTM consolidation depends on the morphological establishment, maintenance, and rearrangement of specific neural networks, which includes the strengthening of synapses or the formation of new connections within specific brain areas involved in learning and memory [1–4]. Importantly, concurrently with morphological changes of synaptic connections, transient induction of new gene transcription and protein synthesis are required for LTM formation [5–7]. Indeed, it has been shown that pharmacological blockade of transcription or translation, as well as the targeted mutation of transcription and translation factors, inhibits LTM consolidation [8–11].

One limitation with the current model used to explain LTM consolidation is that at the cellular level synaptic connections and electric patterns are highly dynamic and unstable, while memories can endure for months, years, and even decades. Similarly, at the molecular level, mRNA and proteins undergo molecular turnover. Some have suggested that epigenetic modulation may explain the permanence of memories [12–15]; however, histone modifications are highly dynamic and reversible [16, 17]. In addition, the rapid turnover rate of transcriptionally active chromatin is a common feature in all nonproliferating cells, including neurons [18–20]. Similarly, DNA methylation and demethylation are dynamic and reversible even in nondividing cells, such as neurons [17, 21]. Hippocampal DNA methylation changes following learning are rapid, but these changes are plastic, not permanent [14, 22]. Moreover, epigenetic mechanisms that chemically modify histones or genomic DNA both regulate transcription. Hence, epigenetic mechanisms most probably function by temporarily regulating transcription during memory and plasticity processes.

In order to assess these questions, our group, as well as others, has been carrying out alternative studies in order to evaluate the possible role of other potential mechanisms that might also be relevant to LTM consolidation. Specifically, we initially postulated that mechanisms involved in DNA recombination/repair may contribute to LTM processes [23]. In the immune system, DNA recombination of gene segments is a well-controlled process involving the activation of DNA endonucleases, which in turn generate DNA DSBs, as well as activation of DNA ligases and DNA repair factors for rejoining new gene segments [24–26]. Interestingly, a recent study reported that when mice explored a novel environment, DNA DSBs were accumulated throughout the brain, particularly in the hippocampus, a region involved in learning and memory [27]. Moreover, these DNA DSBs were repaired within 24 h, suggesting that a physiological machinery for the introduction and repair of these DNA lesions may be related to learning and memory processes. Furthermore, subsequent studies reported that DNA DSBs are introduced in the promoters of a subset of immediate early genes including Fos, Npas4, and Egr1 in response to neuronal activity, synaptic plasticity processes, and learning [28]. Consistent with these findings, we previously reported that Fen-1 endonuclease [29], terminal deoxynucleotidyl transferase (TdT), a template-independent DNA polymerase involved in V(D)J recombination [30], DNA ligase [31, 32], and Non-Homologous End Joining (NHEJ) activity [32, 33] are DNA recombination/repair factors or machineries regulated by and/or required for learning and memory processes.

Here, we report that the* V(D)J recombination-activating gene 1* (*RAG1*), which is key to initiating V(D)J recombination in lymphocytes [34–37], is a factor modulated by context fear conditioning in young adult mice and that its amygdalar expression is required for LTM. Quantitative real-time PCR indicated that* RAG1* mRNA is induced in the amygdala, but not in the hippocampus, after conditioning. Such induction is related to associative learning, rather than to the nonassociative behavioral experiences related to context fear conditioning, as determined with Naïve, context-only, and shock-only controls. Additional control experiments confirmed the sequence identity between amygdalar and thymus* RAG1* PCR products, both showing 100% match to* Mus musculus RAG1* in BLAST analyses. Moreover, double immunofluorescence studies indicated that RAG1 protein is expressed within amygdalar neurons. The functional relevance of* RAG1* was examined using gapmer antisense, versus random oligonucleotides infused directly into the amygdala either immediately prior to or 5 h after conditioning. Pretraining infusions resulted in amygdalar knockdown of* RAG1* mRNA and a significant impairment in LTM, while posttraining infusions did not affect LTM. Together, these findings suggest that* RAG1* plays a role in LTM consolidation.

## 2. Materials and Methods

The Institutional Animal Care and Use Committee (IACUC) of the Río Piedras Campus of the University of Puerto Rico in compliance with National Institutes of Health (NIH) guidelines for the care and use of laboratory animals (Department of Health and Human Services NIH publication number 86-23) approved all procedures involving animals.

### 2.1. Contextual Fear Conditioning

#### 2.1.1. Apparatus

Our conditioning chamber (30 × 20 × 18 cm) was made of transparent Plexiglas on two sides and stainless steel on the other two sides. Each of the steel sides had a speaker and a 24 V light. The chamber had a 36-bar-insulated shock grid floor made of stainless-steel rods (Coulbourn Instruments, Allentown, PA). The system included a white-noise generator to provide background noise (70 dB). The floor was removable and was cleaned with 70% ethanol after each subject was trained, reexposed, or tested. Each bar (1.5 cm in diameter) was connected through a harness to a programmable Master Shocker (model 82404SS; Coulbourn Instruments) that delivered scrambled foot shocks to each of the bars in the grid floor. A mini camera (Silent Witness Enterprises, Surrey, British Columbia, Canada) installed directly behind one of the two Plexiglas sides of the conditioning chamber was connected via a processor to a computer system for video recording and scoring of freezing using the Xpress SDK software, which is a PCI bus mastering wavelet video compression/decompression and capture board (Integral Technologies, Indianapolis, IN).

#### 2.1.2. Subjects and Training

Context conditioning was done essentially as previously described [11, 32, 38]. Male C57BL/6 mice of 8–10 weeks of age from Harlan Sprague Dawley, Indianapolis, IN, were used. Food and water were available at all times, and the animals were kept on a 12 h light/dark cycle. In contextual fear conditioning, animals were placed in the conditioning chamber (conditioned stimulus, CS) and allowed to explore for 2 min (habituation). Animals then received three foot shocks of 0.75 mA for 2 s (unconditioned stimulus, US) delivered at 2, 3, and 4 min. Mice remained in the chamber 30 s after the last shock and were then immediately moved to their home cages.

### 2.2. RNA Extraction, Quantification, and Quality Evaluation

Once trained, animals were decapitated at 15 min, 30 min, or 1 h after conditioning. Some animals were also used as Naïve, 15 min context-only (CO) and 15 min shock-only (SO) controls. CO mice were exposed to the conditioning context for 4 m without receiving any shocks and SO mice received a rapid single shock and were immediately removed from the conditioning chamber. After removal from the conditioning chamber, both CO and SO mice were returned to their homecages and sacrificed 15 min after the end of their exposure to the conditioning chamber. Brains were rapidly obtained, chilled in ice-cold Phosphate Buffered Saline (PBS), and then transferred to a mouse brain matrix to obtain bilateral amygdalar tissue punches and dissection of the dorsal hippocampus, both between −0.82 and −2.70 mm from bregma points, based on the mouse brain in stereotaxic coordinates, third edition [39]. All tissues were kept in RNA*later* (Ambion, Cat. number 7020) solution on dry ice and later stored at −86°C until RNA extraction. RNA was extracted from individual dorsal hippocampi using the Qiagen RNeasy Mini Kit (Cat. number 74104), while bilateral amygdalar punches were RNA extracted using the Qiagen RNeasy Micro Kit (Cat. number 74004). Extracts were treated using DNase (Qiagen, 79254) and the kit's protocol was performed. RNA samples were quantified using the NanoDrop ND-1000 spectrophotometer and quality was evaluated using the Agilent 2100 Bioanalyzer system.

### 2.3. Quantitative Real-Time PCR

#### 2.3.1. Primer Design for Real-Time PCR

*cDNA* sequences from* Mus musculus* genes analyzed [*RAG1*, accession number NM_009019.2; and* glyceraldehyde 3-phosphate dehydrogenase* (*gapdh*), accession number NM_008084.2] were obtained from GenBank. We used the Integrated DNA Technologies PrimerQuest and Oligo Analyzer bioinformatics tools to design specific primers suitable for real-time PCR and also avoid possible hairpins, self/homodimers, and hetero/cross-dimers. A BLAST (basic local alignment search tool) search was done on all primers to ensure that they would not potentially anneal to other targets. The following forward and reverse primers were used:* RAG1*, forward, 5′-TGA GCA CAG GCA AGC TGA TGA-3′ and* RAG1* reverse, 5′-TTG ACA CGG ATG GCC AAG CAA-3′; for* gapdh* forward 5′-ACC CAG AAG ACT GTG GAT GG′-3 and* gapdh* reverse 5′-ACA CAT TGG GGC TAG GAA CA-3′. All primers were synthesized by Integrated DNA Technologies.

#### 2.3.2. cDNA Synthesis and Quantitative Real-Time PCR

Briefly, cDNA was synthesized using the TaqMan Reverse Transcription (RT) Reagents kit (N8080234, Applied Biosystems/Roche). 250 ng of RNA, 2.5 *μ*L of RTBuffer (10x), 6.0 *μ*L MgCl_2_ (25 mM), 5.0 *μ*L dNTPs (10 mM, 2.5 mM, each nucleotide), 2.0 *μ*L OligodT (50 *μ*M), 0.5 *μ*L RNase inhibitor (20 U/*μ*L), and 3.0 *μ*L RT Enzyme (50 U/*μ*L) in a total volume of 25 *μ*L were used. Thermal cycler conditions were as follows: 25°C for 10 min, 48°C for 30 min, 95°C for 5 min, and 4°C forever. Real-time PCR was performed using the QuantiTect SYBR Green PCR Master Mix Kit (Qiagen, 204163). Real-time PCR amplification conditions were optimized for each gene and we obtained the best results under the following master mix conditions: for housekeeping control gene* gapdh* we used 12.5 *μ*L of SYBR Green Master Mix, 2.5 *μ*L of each primer (5 *μ*M), and 2 *μ*L of cDNA in 25 *μ*L of reaction. For* RAG1*, we used 12.5 *μ*L of SYBR Green Master Mix, 3.5 *μ*L of each primer (5 *μ*M), 1.5 *μ*L of MgCl_2_ (25 mM), and 2 *μ*L of cDNA in 25 *μ*L of reaction. Real-time PCR amplifications were run in triplicate for each gene per sample in a thermal cycler (Bio-Rad C1000 Touch CFX96 Real-Time PCR System). Amplification conditions were optimized for both genes at 95°C 15 min (hot start), followed by 40 cycles at 95°C for 15 s, 30 s at 58°C, and 72°C for 30 s. Finally, a melt/peak curve analysis was performed from 55°C to 95°C at increasing temperature rate of 0.5 degrees.

#### 2.3.3. Real-Time PCR Analysis

We used the comparative threshold cycle (Delta Ct) method of relative quantification to calculate gene expression levels [40]. Delta Ct method involves comparing the Ct values of the genes of interest with a reference or housekeeping gene: Ct = Ct Target − Ct Reference gene (Ct_t_ − Ct_r_). In this case, the Ct value of* RAG1* primers' amplification product is subtracted from the Ct of* gapdh* primers' amplification product (Ct_*RAG*1_ − Ct_*gapdh*_). First, the Ct-average of triplicates per gene per sample from PCR amplification was obtained. Importantly, our analyses only used samples displaying triplicate results with high reproducibility, that is, those showing triplicate differences in Ct of no more than 0.05%. In the case that the real-time PCR experiments of a particular sample resulted in low reproducibility, such specific experiments were repeated or in some cases discarded, always making sure that the final analyses could be made with the result of triplicate reactions per sample. As Ct is proportional to the logarithm of initial amount of target in a sample, the relative concentration of one target contextual fear conditioning-trained with respect to a reference (Naive) is reflected in the difference in cycle number (Ct) necessary to achieve the same level of fluorescence. Delta Ct data is then normalized by 2^−(deltaCt  trained − deltaCt  naive)^.

### 2.4. Amplification and Molecular Cloning of* RAG1* PCR Products

We amplified a* RAG1* mRNA fragment of approximately 48 bp (the same from the real-time PCR experiments described above) from amygdalar, hippocampus, and thymus tissue and also cloned the resulting PCR products for sequence analysis. Briefly, following RNA extraction and purification (as above), cDNA was synthesized using TaqMan Reverse Transcription Reagents kit (N8080234, Applied Biosystems/Roche). 500 ng of RNA from each sample was used and mixed with 2.5 *μ*L of RT Buffer (10x), 4.5 *μ*L MgCl_2_ (25 mM), 4.0 *μ*L dNTPs (10 mM, 2.5 mM each nucleotide), 2.0 *μ*L OligodT (50 *μ*M), 0.5 *μ*L RNase inhibitor (20 U/*μ*L), and 3.0 *μ*L of RT Enzyme (50 U/*μ*L) in a total volume of 25 *μ*L. cDNA synthesis was performed in a thermal cycler (GeneAmp PCR System 9700, Applied Biosystems) under the following conditions: 25°C for 10 min, 48°C for 30 min, 95°C for 5 min, and 4°C forever. PCR was performed using the PCR Master Mix (2x) (2 × 50 reactions) Kit (Promega, M7502). PCR amplification conditions were performed under the following master mix conditions: 12.5 *μ*L of master mix, 3.5 *μ*L of each primer (5 *μ*M, the same primers used for real-time PCR experiments), 1.5 *μ*L of MgCl_2_ (25 mM), and 2 *μ*L of cDNA in 25 *μ*L of reaction. Amplifications were performed by an initial denaturation at 95°C for 30 s, followed by 40 cycles at 95°C for 15 s, 58°C for 30 s, and 72°C for 30 s, and final extension at 72°C for 7 min followed by 4°C forever. PCR amplifications were evaluated by agarose (1%) gel electrophoresis in TAE1X and stained with ethidium bromide. PCR products were purified from the gel using the QIAquick Gel Extraction Kit (50), following the kit protocol manual (Qiagen, 28704). Purified PCR products were cloned using the pGEM-T Easy Vector System I (Promega, A1360). DNA ligation and plasmid transfection were performed as recommended by the kit's protocol. For plasmid transfection we used* E. coli* (from Invitrogen, 18258-012) MAX Efficiency DH5*α* Competent Cells. Cells were plated onto LB/ampicillin agar medium containing IPTG and X-Gal.

### 2.5. Plasmid DNA Extraction and Purification for Sequencing

Minipreps for sequencing were performed using a modification of standard methods [41]. Briefly, a single white bacterial colony was transferred into 3 mL of Terrific Broth medium containing ampicillin in a loosely capped 15 mL tube. Cells were cultured overnight at 37°C with vigorous shaking. Harvest and extraction was performed as indicated in standard methods [41], but we also did additional successive purification steps, including RNase A treatment, exonuclease digestion, and proteinase K incubation. After two chloroform extractions and isopropanol precipitations, pellets were dried, dissolved in 50 *μ*L of H_2_O, and mixed with 12 *μ*L of NaCl 4 M and 60 *μ*L of freshly made polyethyleneglycol 13% (PEG 8000). The mixture was incubated on ice for 30–60 min and centrifuged for 15 min and the pellet was rinsed with ethanol 70%. Finally, pellets were dried and dissolved in 50 *μ*L of H_2_O. Samples were kept in −20°C until used. Sequencing reactions were performed using the BigDye Terminator Chemistry v.3 in an ABI 3130xl Genetic Analyzer. DNA sequences were analyzed by BLAST against the mouse genome (RefSeq RNA) and aligned using ClustalW2.

### 2.6. Histology: Brain Perfusion and Tissue Preparation

One hour after context fear conditioning, animals received a lethal dose of avertin and were immediately followed by transcardial gentle perfusion with PBS buffer for 5 min. After that, we switched to the fixing buffer (4% paraformaldehyde, pH 7.4) for 5 min. Brains were extracted, fixed in 4% paraformaldehyde solution for 24 h, and later cryoprotected overnight in a 30% sucrose solution and finally frozen at −86°C for storage until future use. Frozen brains were used to collect 20 *μ*m thick coronal sections selecting specifically those containing the amygdala region between −1.06 and −2.30 mm bregma points, based on the mouse brain in stereotaxic coordinates, third edition [39]. The collected sections were placed onto positively charged slides (Probe-On Plus Slides, Fisher, PR) and kept at −86°C until used for immunofluorescence.

### 2.7. Immunofluorescence

To examine the cellular localization of RAG1 protein expression, slides were dried up at rt for 30 min. Using a heat block set at 200°C, a beaker flask containing the buffer (800 mL ddH_2_O, 4 mL 1 M Tris pH 8, and 1.6 mL 0.5 M EDTA) was heated until boiling. Once the buffer's bubbles were moving slowly, slides were slowly immersed into the buffer using a slide-rack. The beaker was immediately covered with a saran wrap containing holes punched with a pipet tip and was allowed to sit for 20 min. The beaker containing the slides was then transferred into an ice bath and allowed to cool down in a cold room (4°C) for 30 min. Slides were partially dried and borders drawn with PAP-Pen. Then, 250 *μ*L of blocking solution (BS) (1x PBS, 10% normal goat serum, and 0.1% Tween-20) was added per slide and incubated for 1 h at rt. Double immunofluorescence was performed, incubating the sections with primary rabbit polyclonal antibody against human RAG1 (Sigma-Aldrich: SAB2106610) diluted at 1 : 100 in 1% goat serum/PBS together with primary anti-NeuN mouse monoclonal antibody (Millipore, MAB377) diluted at 1 : 100. Incubation with primary antibodies was done overnight at 4°C in a moist chamber. Slides were washed with PBS 3 times for 5 min each. Slides were then incubated for 2 h at rt in the dark moist chamber with Alexa Fluor 488-conjugated goat anti-rabbit IgG secondary antibody (Invitrogen) (for detection of RAG1) and Alexa Fluor 568-conjugated goat anti-mouse IgG secondary antibody (Invitrogen) (for detection of NeuN), both diluted at 1 : 200 in 1% goat serum/PBS, followed by 3 PBS washes for 5 min each. Slides were mounted using permanent mounting medium (Vector Laboratories). All slides were first scanned at low magnification (10x) to locate the amygdala, which was subsequently analyzed at higher magnification (40x) using a Zeiss LSM-5 Pascal scanning confocal microscope. Final image composites were created using Zeiss LSM5 PASCAL Image software, version 3.2.

### 2.8. Protein Extraction

Mice were decapitated 1 h after training and their brains were obtained, chilled on ice-cold PBS, and used to dissect the amygdala as previously explained, between −0.82 and −2.7 mm bregma points, based on the mouse brain in stereotaxic coordinates [39]. Bilateral amygdalar tissue punches from three animals were combined yielding one pool sample per group. Thymus, bone marrow, and muscle tissues were also dissected as controls. Tissues were stored at −86°C until used for protein extraction. Protein extracts were prepared as described by us previously [29, 31–33]. Briefly, tissues were homogenized using a sonic dismembrator in extraction buffer [30 mM HEPES/KOH, pH 7.9, 0.5 M KCl, 5 mM MgCl_2_, 1 mM EDTA, 2 mM dithiothreitol (DTT), 20% glycerol, 1 mM phenylmethylsulfonyl fluoride (PMSF), and 1 *μ*g/mL of each of leupeptin and aprotinin] and incubated for 1 h on ice. Extracts were centrifuged at 14,000 rpm for 1 h at 4°C. The supernatant was then dialyzed for 4 h in dialysis buffer (30 mM HEPES/KOH, pH 7.9, 50 mM KCl, 2 mM EDTA, 5 mM MgCl_2_, 1 mM DTT, 10% glycerol, 1 mM PMSF, and 1 *μ*g/mL of each of leupeptin and aprotinin). Dialyzed fractions were centrifuged at 14,000 rpm for 30 min at 4°C. Protein extracts were stored at −86°C until used for Western blots. The protein concentration was determined in the NanoDrop ND-1000 spectrophotometer.

### 2.9. Western Blotting

For Western blotting, protein samples (50 *μ*g) and 4 *μ*L of the Odyssey Prestained Molecular Weight Marker (LI-COR Biosciences, Nebraska, USA) were first separated on a 8% sodium dodecyl sulfate-polyacrylamide gel electrophoresis (SDS-PAGE). The separated proteins in the gels were transferred to a nitrocellulose membrane using a semidry electroblotter system at 15 V for 1 h and 30 min. Then, the membranes were blocked overnight using a mixture of 5% nonfat milk and Odyssey Blocking Buffer (LI-COR Biosciences, Nebraska, USA) on an orbital shaker. After 3 washes of 15 min each with PBS Tween-20 (PBS-T), the membrane was incubated with a mixture of two primary antibodies: 1 : 2,000 dilution of a rabbit polyclonal antibody raised against a synthetic nonphosphopeptide derived from human RAG1 (Sigma-Aldrich: SAB2106610) and 1 : 3,000 dilution of a mouse monoclonal anti-actin antibody (Sigma-Aldrich: A4700) at 4°C overnight. After 3 washes of 15 min each with PBS-T, the membranes were incubated with a mixture of two secondary fluorescent antibodies (1 : 10,000 dilution of donkey anti-rabbit IRDye680 and 1 : 12,000 dilution of donkey anti-mouse IRDye800) (LI-COR Biosciences, Nebraska, USA) for 2 h at rt. The membranes were washed 3 times as previously mentioned and scanned for analysis using the Odyssey Infrared Imaging System.

### 2.10. Antisense Knockdown Experiments

#### 2.10.1. RAG1 Antisense Oligonucleotides for Gene Knockdown

Gapmer antisense oligonucleotides were designed to target the start codon of mouse* RAG1* mRNA. This 20 bp gapmer contains a central block consisting of ten phosphorothioate-deoxynucleotides, sufficient to induce RNaseH cleavage (and thus targeted RNA:DNA hybrid degradation), and it is flanked by two blocks, each consisting of five 2′-*O*-methyl-modified ribonucleotides that protect the internal phosphorothioate-deoxynucleotide block from nuclease degradation, thus increasing stability. As a control, a random sequence was also designed with the same backbone modifications and base composition as the* RAG1* antisense, but in a scrambled sequence order and without homology to any known mouse gene.* RAG1* antisense and random oligonucleotide sequences were, respectively, as follows: 5′-mGmCmC mAmCA^*∗*^  G^*∗*^A^*∗*^G^*∗*^  A^*∗*^T^*∗*^A^*∗*^  G^*∗*^C^*∗*^A^*∗*^ mAmCmA mUmA-3′ and 5′-mCmAmG mAmUA^*∗*^  A^*∗*^C^*∗*^C^*∗*^  G^*∗*^T^*∗*^A^*∗*^  G^*∗*^A^*∗*^G^*∗*^ mCmAmA mCmA-3′ (where “m” represents 2′-*O*-methyl RNA and “*∗*” represents phosphorothioate DNA). Antisense and random sequences were synthesized by Integrated DNA Technologies. All batches of antisense and random oligonucleotides used to complete these studies were received lyophilized and fully purified by RNase Free HPLC. Oligonucleotides were dissolved in sterile 1x TE buffer pH 7.5 solution to a final concentration of 0.2 nmol/*μ*L.

#### 2.10.2. Surgeries

Several surgeries were conducted to determine the proper coordinates for implantation of cannulae into the amygdala, based on the mouse brain in stereotaxic coordinates [39]. For surgery, animals were anesthetized with avertin and placed into a stereotaxic apparatus (David Kopf Instruments), with the nose angled at 0°. After a scalp incision was made, lambda and bregma were located, and holes were drilled in the skull above the target region. Bilateral cannulae (23 gauges) guide (6.8 mm wide) was implanted above the amygdala in order to avoid damaging the amygdala tissue complex with cannulae and injectors while at the same time ensuring amygdala-enriched distribution. The following coordinates were used: anterior-posterior, +1.0 mm from bregma; medial-lateral, −3.4 mm from midline; and dorsoventral, −2.5 mm from skull. The cannulae were secured to stainless-steel screws with dental cement and a light-curable resin. Wire stylets (33 gauges) were inserted into the cannulae guides and checked every day to ensure clean and functional cannulae.

#### 2.10.3. Intra-Amygdalar Oligonucleotide Microinfusions, Behavioral Training, and Memory Testing

After surgery, animals were given a two-day period to recover. Afterwards, animals were handled during 3 days, 3 min each. The fourth day, animals were bilaterally microinfused with 1 *μ*L of 1x TE buffer pH 7.5 (2 min at 0.5 *μ*L/min), as a handling/stress control, and then we proceeded with the regular 3 min manual handling. Microinfusions were accomplished by inserting a 33-gauge stainless-steel injector into the guide cannulae so that it extended 1 mm beyond the tip of the guide, right above the targeted amygdalar complex (see above). Functionality of injectors was verified before every microinfusion between animals and replaced when necessary. After infusion, the injectors were removed, and the stylets were replaced. The fifth day, animals were bilaterally microinfused (2 min at 0.5 *μ*L/min) with 0.2 nmol of* RAG1* antisense or random sequence oligonucleotides, handled for 3 min, and returned to their home cages. One hour after the microinfusions, animals were trained in context fear conditioning and video recorded as described above. One day (24 h) after training mice were reintroduced into the same conditioning context for LTM testing by measuring freezing in the video recordings for 4 m, but they did not receive shocks. For the posttraining injection studies, a different set of animals was treated as above except that the antisense or random oligonucleotide treatment was given 5 h after context fear conditioning instead of 1 h prior to training. For the reconsolidation studies, another set of mice was subjected to surgery cannulation and received handling as above, but in the fifth day, mice were microinfused with saline (as a stress control) and trained in context fear conditioning 1 h later. Next, 24 h after conditioning, animals were microinfused with either random or antisense oligonucleotides 1 h prior to reexposure to the conditioning chamber and video recorded for 90 s for memory retrieval without receiving any shocks. Finally, 48 h after conditioning (24 h after context reexposure), mice were again reexposed to the conditioning chamber for 2 min to measure freezing of LTM in the reconsolidation test.

#### 2.10.4. Diffusion Studies

After cannulae implantation, injectors were inserted and animals (*n* = 4) were infused with FITC-*RAG1* antisense oligonucleotides to estimate the area of the antisense diffusion within the amygdala. An infusion of 1 *μ*L of FITC-*RAG1* antisense oligonucleotide (0.2 nmol) was delivered bilaterally into the amygdala during a 2 min period at a rate of 0.5 *μ*L/min. Animals were decapitated 3 h after infusion, and their brains were isolated and stored at −86°C. Coronal amygdalar sections, 20 *μ*m thick, were scanned at low magnification (10x) to locate the amygdala, which was subsequently analyzed at higher magnification (20x and 40x) using a Zeiss LSM-5 Pascal scanning confocal microscope. Images were processed using Zeiss LSM5 PASCAL Image software, version 3.2.

### 2.11. Statistical Analysis

All statistical analyses were performed with Prism 4 software (GraphPad Software). Statistical significance was assumed at *P* < 0.05. Real-time PCR experiments of* RAG1* mRNA levels were analyzed by One-Way ANOVA and Newman-Keuls posttests to compare* RAG1* mRNA expression between behavioral groups. Real-time PCR experiments of perfused and nonperfused mice for* RAG1* levels were analyzed using Student's *t*-test. Knockdown validation of* RAG1* antisense mRNA levels in the amygdala compared to random was analyzed by Student's *t*-test. Memory acquisition behavioral data was subjected to Two-Way Repeated Measures (RM) ANOVA and Bonferroni posttesting. LTM behavioral tests analyses were analyzed by Student's *t*-test. Memory retrieval and reconsolidation tests were analyzed by Two-Way ANOVA coupled to Bonferroni posttesting.

## 3. Results

### 3.1. Context Fear Conditioning Learning Specifically Induces* RAG1* mRNA Expression in the Amygdala

**Figure 1:**
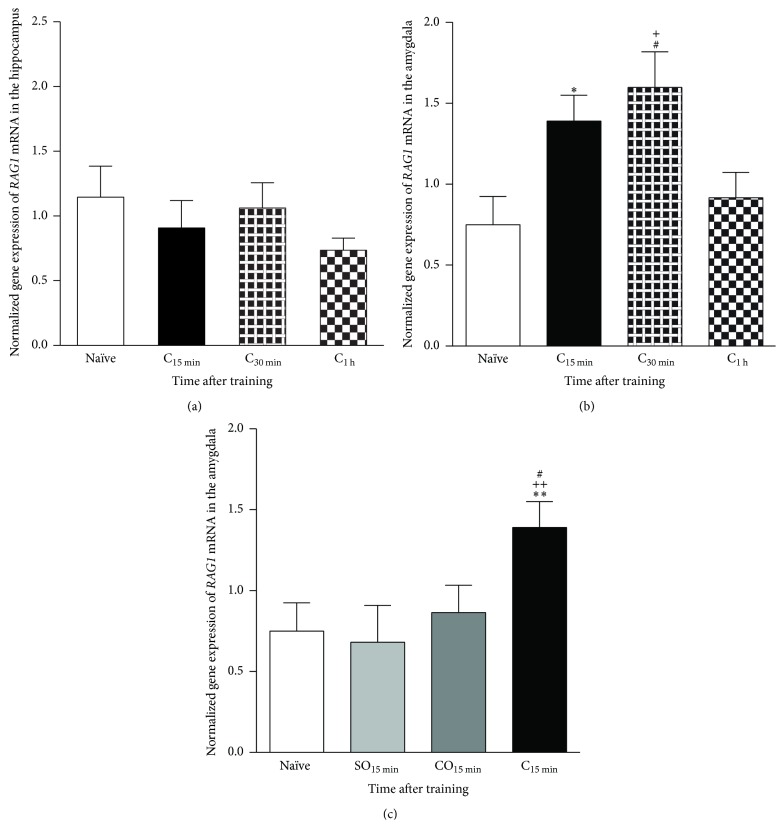
Context fear conditioning induced upregulation of* RAG1* mRNA in the amygdala.* RAG1* mRNA levels were measured in the hippocampus and the amygdala on a time course at 15 min, 30 min, and 1 h after conditioning. (a) Normalized mRNA data showed no significant differences when examining hippocampal* RAG1* mRNA Naïve (N) or the conditioned (C) groups sacrificed at 15, 30, or 60 min after training. (b) In contrast, context fear conditioning results in a significant, rapid, and transient induction in* RAG1* mRNA levels in the amygdala (Naïve versus C15 min, ^*∗*^ *P* < 0.05; Naïve versus C30 min, ^#^ *P* < 0.05; Naïve versus C60 min, *P* > 0.05; C60 min versus C30 min, ^+^ *P* < 0.05; and C60 min versus C15 min, *P* > 0.05). (c) We sacrificed animals from the conditioned (C), CO, or SO groups 15 min after their respective associative or nonassociative training. Normalized expression confirmed the significant induction at 15 min of amygdalar* RAG1* mRNA after context fear conditioning compared to Naïve, CO, and SO groups and showed no statistical difference between Naïve, CO, or SO controls (SO15 min versus C15 min, ^#^ *P* < 0.05; SO15 min versus Naive, *P* > 0.05; SO15 min versus CO15 min, *P* > 0.05; CO15 min versus C15 min, ^++^ *P* < 0.01; CO15 min versus Naive, *P* > 0.05; and Naïve versus C15 min ^*∗∗*^ *P* < 0.01).

DNA recombination/repair processes involve the activation of endonucleases as well as DNA ligases and polymerases, among other factors. We focused our present studies on* RAG1*, the gene encoding the specialized recombinase of V(D)J recombination, which initiates V(D)J recombination* in lieu* of its site-specific endonuclease activity that targets highly specific recombination signal sequences (RSSs) introducing DNA DSBs in antigen receptor genes [34, 36, 37, 42] and which we identified in a preliminary DNA microarray screen as a potential candidate gene involved in context fear conditioning. For the experiments reported here, we initially trained C57BL/6 male mice in context fear conditioning, sacrificed at 15, 30, or 60 min after training, and obtained dorsal hippocampi and amygdalar tissues. We used quantitative real-time PCR to amplify a fragment of* RAG1* mRNA and determine whether the expression of this gene is modulated in the amygdala or the hippocampus in association with context fear conditioning learning. The results of these experiments are shown in Figure 1. As seen in Figure 1(a), when examining hippocampal* RAG1* mRNA no significant differences were observed between the Naïve (Naive, *n* = 6) and the conditioned (C) groups sacrificed at either 15 (*n* = 7), 30 (*n* = 7), or 60 (*n* = 7) min after training (One-Way ANOVA: *F*(3,23) = 0.8966, *P* > 0.05). The results demonstrate that the basal levels of hippocampal* RAG1* mRNA do not change significantly after training. In contrast to our findings with the hippocampus, we found that context fear conditioning training results in a significant, rapid, and transient induction in* RAG1* amygdalar mRNA levels (Figure 1(b): One-Way ANOVA, *F*(3,30) = 4.753, ^*∗∗*^ *P* < 0.01; Multiple Comparison Testing: Naïve versus C15 min, ^*∗*^ *P* < 0.05; Naïve versus C30 min, ^#^ *P* < 0.05; Naïve versus C60 min, *P* > 0.05; C60 min versus C30 min, ^+^ *P* < 0.05; and C60 min versus C15 min, *P* > 0.05). Overall, these results show that* RAG1* mRNA is induced rapidly and transiently after context fear conditioning training in the amygdala, but not the hippocampus, of young adult C57BL/6 mice. For our next set of experiments, we aimed to determine if such amygdalar induction is specific to associative context fear conditioning, using nonassociative context-only (CO, *n* = 6) and shock-only (SO, *n* = 8) controls, in which the conditioned stimulus (CS) (for CO controls) or unconditioned stimulus (US) (for SO controls) was presented individually, rather than paired. We sacrificed animals from the C (*n* = 9), CO, or SO groups 15 min after their respective associative or nonassociative training. Brains were obtained, amygdalar tissue punches were dissected, and RNA was extracted. Naïve animal controls were also used (Naive, *n* = 8). The results can be seen in the bar graph in Figure 1(c). The results again confirmed the induction at 15 min of amygdalar* RAG1* mRNA after context fear conditioning compared to Naïve, CO, or SO controls and showed no statistical significant difference between any of these controls (One-Way ANOVA, *F*(3,27) = 5.943, ^*∗∗*^ *P* < 0.005; Multiple Comparison Testing: Naive versus C15 min, ^*∗∗*^ *P* < 0.01; SO15 min versus C15 min, ^#^ *P* < 0.05; CO15 min versus C15 min, ^++^ *P* < 0.01; SO15 min versus Naive, *P* > 0.05; SO15 min versus CO15 min, *P* > 0.05; CO15 min versus Naive, *P* > 0.05). Finally, since the results presented so far were obtained from tissue samples obtained from nonperfused brains, we carried out an additional control experiment to determine if the observed changes in* RAG1* mRNA levels could be attributed to the presence of blood cells in the brain or not, since immune cells in the blood are a known biological site of* RAG1* expression [43, 44]. We trained two groups of mice in context fear conditioning and sacrificed animals of both groups 30 min after training, a time corresponding to the peak for amygdalar* RAG1* mRNA induction observed after training (see Figure 1(b)). The brains of animals in one group were obtained as described above. For the second group of animals, mice were injected with a lethal dose of avertin 25 min after training and perfused for 3 min with PBS-1X in order to remove the blood from their brains. Brains from both groups of animals were extracted and amygdalar tissues (*n* = 4 nonperfused; *n* = 4 perfused) were dissected. Amygdalar tissues were used for further RNA isolation, cDNA synthesis, and quantitative real-time PCR analysis of* RAG1* mRNA. The results showed no significant difference between the levels of amygdalar* RAG1* mRNA 30 min after context fear conditioning training of nonperfused (1.304 ± 0.3377) or perfused (1.274 ± 0.2751) mice (Student's *t*-test; *t*_(7)_ = 0.1473; *P* > 0.8), ruling out the possibility that the observed induction of* RAG1* mRNA (see Figure 1(b)) could be due to residual blood, and thus blood cells, in the examined brain tissues.

### 3.2. Sequence Analysis of PCR Products Amplified from the Hippocampus, Amygdala, and Thymus

We next set out to confirm the sequence identity of the PCR products from amygdalar and hippocampal tissues amplified using the set of primers designed to target* RAG1* mRNA in our experiments presented above. In addition to amygdalar and hippocampal RNA samples, we also utilized thymus RNA, since this tissue is known to physiologically express* RAG1* due to its role in the immune system [45–48]. Supplementary Figures  1A and B (in Supplementary Material available online at http://dx.doi.org/10.1155/2016/1752176) depict the representative results of amplification curves, as well as our melting curve analysis, respectively, for* RAG1* and* gapdh* mRNAs. Results of amplification and melting temperature curves of* RAG1* and* gapdh* are depicted to show the cycle thresholds (Ct) for both genes and the specificity of the amplification products, respectively. Importantly, the results of the melting curve analyses consistently demonstrated that only one specific product per primer-gene set was generated. The* RAG1* amplification products amplified by standard PCR were also visualized by agarose gel electrophoresis (Supplementary Figure  1C). Cloning and sequencing of these PCR products amplified from amygdala, hippocampus, and thymus confirmed their identity as* RAG1* (Supplementary Figures  2D–F) or* gapdh* (data not shown). Sequencing electropherograms from* RAG1* PCR products are shown in Supplementary Figure  2D. PCR fragment sequences from amygdala, hippocampus, and thymus were aligned using ClustalW2 (Supplementary Figure  2E) and compared with* Mus musculus RAG1* reference sequence NM_009019.2 (Supplementary Figure  2F). BLAST analysis confirmed the molecular identity of* RAG1* PCR products from amygdala, hippocampus, and thymus showing 100% matched identity to* Mus musculus RAG1* (Ref|NM_009019.2) in mouse genome BLAST analyses with an *E*-value of 2e − 19.

### 3.3. RAG1 Protein is Localized within Amygdalar Neuronal Cells

**Figure 2:**
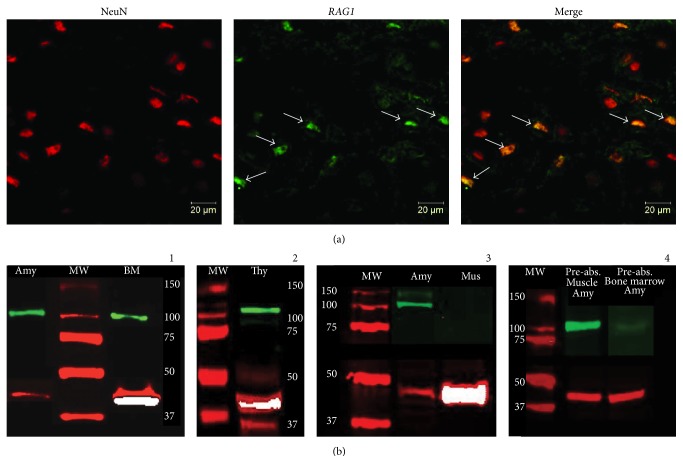
RAG1 protein expression in amygdalar neuronal cells. Amygdalar coronal sections of context fear conditioning-trained mice, perfused 1 h after conditioning, were used for immunofluorescence and analyzed by confocal microscopy. Antibodies from immunofluorescence were validated by Western blot analysis. (a) Amygdalar area representative images of a double immunostaining using RAG1 antibody labeled with Alexa Fluor 488, green channel signal, and NeuN antibody labeled with Alexa Fluor 568, red channel signal. The left panel shows the NeuN positive neuronal nuclei, while the middle panel depicts RAG1 immunopositive cells. The right panel is the merge image showing colocalization of the NeuN neuronal nuclei marker and RAG1. Arrows point to some of the RAG1 immunopositive neurons. These immunofluorescent images revealed colocalization of RAG1 protein expressing cells with those expressing NeuN, suggesting the presence of RAG1 in neurons, although not all neurons expressed RAG1. (b) Tissue punches from amygdala (Amy) were obtained 1 h after context fear conditioning and analyzed in Western blot by comparative comigration with a standard molecular weight (MW) marker and protein extracts from bone marrow (BM) ((b)-1) and thymus (Thy) ((b)-2). Both sets of experiments consistently showed comigration between the tissues with a band corresponding to ~120 KD of RAG1 protein (green channel corresponding to RAG1 and red channel corresponding to beta-actin, ~42 KD); prestained molecular weight (MW) marker (ladder) was included in all the Western blots. ((b)-3) Additionally, tissue protein extracts from leg muscle (Mus) (negative control) were analyzed compared to amygdalar extracts with respect to RAG1 expression. As expected, RAG1 was not expressed in muscle compared to amygdala ((b)-3), bone marrow ((b)-1), and thymus ((b)-2). ((b)-4) RAG1 antibody preabsorption assays, either with muscle or with bone marrow extracts, showed that only bone marrow extracts, which express RAG1 as opposed to muscle, were able to block the ~120 KD band from amygdalar protein extracts in the Western blots, indicating that RAG1 antibody was preabsorbed (blocked) only by RAG1 protein expressing tissue (bone marrow).

RAG1 is the key endonuclease of V(D)J recombination in immune cells introducing, together with RAG2, DSBs in antigen receptor genes at their RSSs [34, 36, 37, 42]. Previous reports have also suggested that the transcript encoding RAG1 is expressed in nervous cells [34–37, 49]. We therefore considered it important to determine whether RAG1 protein expression is localized to amygdalar neurons. Double immunofluorescence of RAG1 antibody with neuronal nuclear marker, NeuN, was performed on brain coronal sections from animals perfused 1 h after context fear conditioning. Representative images obtained from confocal microscopy examination of the amygdalar areas of brain sections double-labeled with RAG1 (Alexa Fluor 488; green signal) and NeuN (Alexa Fluor 568; red signal) are shown in Figure 2(a). These findings indicate that RAG1 appeared to be expressed predominantly in neurons, as suggested by the colocalization between RAG1 and NeuN. It is also important to mention that while all RAG1 positive cells were neurons, not all neurons showed RAG1 reactivity, suggesting that only a subset of cells expressed RAG1. The molecular specificity of the RAG1 antibody was confirmed by Western blot analyses (Figure 2(b)). Tissue punches from amygdala were obtained 1 h after context fear conditioning. Additionally, reference tissues from thymus and bone marrow, known to express high levels of RAG1 [45–48], and muscle (negative control) were dissected. All tissues were subjected to protein extractions for Western blot analyses. RAG1 protein expression from the amygdala was compared by comigration with bone marrow and standard molecular weight (MW) ladder (Figure 2(b), Panel 1), and thymus (Figure 2(b), Panel 2) extracts, respectively. Both sets of experiments confirmed comigration of a band of approximately 120 KD corresponding to RAG1 protein. In contrast, amygdalar protein extracts compared with muscle extracts (Figure 2(b), Panel 3) showed no comigration of bands indicating, as expected, that RAG1 is specifically expressed in the amygdala (Figure 2(b), Panel 1), as well as bone marrow (Figure 2(b), Panel 1) and thymus (Figure 2(b), Panel 2), but not in muscle tissue (Figure 2(b), Panel 3). Furthermore, RAG1 antibody preabsorption assays with either bone marrow or muscle protein extracts, which display either detectable or undetectable RAG1 expression, respectively (see Figure 2(b), Panel 1 versus Figure 2(b), Panel 3, resp.), showed that only bone marrow protein extracts (known to express RAG1; see Figure 2(b), Panel 1) were able to block the ~120 KD band from amygdalar protein extracts in the Western blots (Figure 2(b), Panel 4). These results indicate that RAG1 antibody was preabsorbed (blocked) only by RAG1 protein expressing tissue (bone marrow).

### 3.4. *RAG1* Plays a Functional Role in LTM Consolidation of Context Fear Conditioning

**Figure 3:**
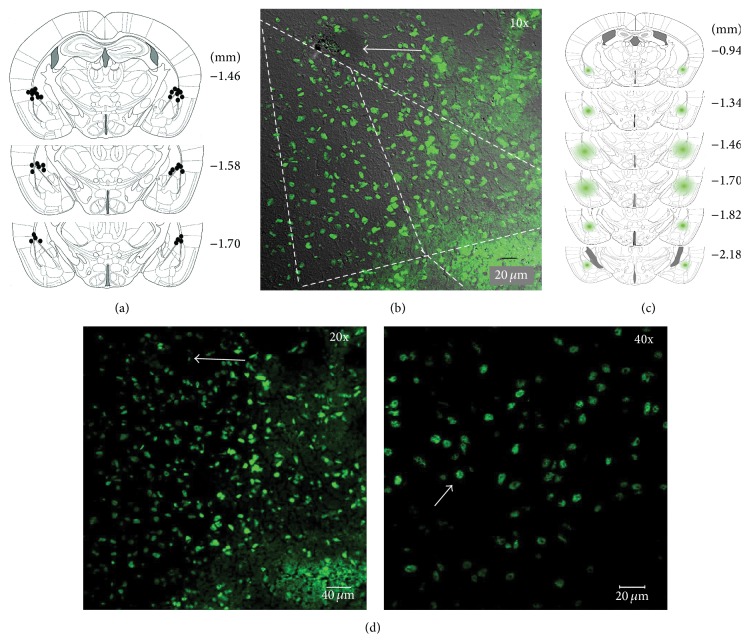
Distribution of cannula placements and* RAG1* antisense oligonucleotide diffusion within the amygdala. After behavioral treatments with* RAG1* antisense or random oligonucleotides, animals were microinfused the next day with thionine to verify cannulae injectors' placement. Another set of animals was used to observe FITC-labeled* RAG1* antisense diffusion. (a) Schematic representation of the amygdala at different rostrocaudal planes illustrating the position of cannulae injectors determined by thionine microinfusion. Injector tips for each cannula are represented by dark spots. (b) FITC-*RAG1* antisense diffusion within the amygdalar complex; arrow indicates the injector's tip. (c) Schemes of coronal sections showing the diffusion of FITC-*RAG1* antisense diffusion into the amygdala of animals decapitated 3 h after fluorescent oligonucleotide infusion. FITC-*RAG1* antisense diffusion is represented by green shading from anterior to posterior areas of the amygdalar complex. The numbers in (a) and (c) indicate the distance from bregma in millimeters. A total of 4 mice were used in these studies. (d) Photomicrograph at higher magnification of FITC-*RAG1* antisense diffusion showed clearly incorporation into the cells (depicted by the arrows) within amygdalar regions.

Our gene expression studies demonstrated that* RAG1* mRNA is specifically induced in the amygdala between 15 min and 30 min as a result of context fear conditioning and it returns to basal levels 1 h after training (see Figure 1(b)). This suggested that RAG1 might play a role in LTM processes. To examine the possible functional role of* RAG1* in LTM consolidation of context fear conditioning, we used an antisense approach to knock down* RAG1* expression in the amygdala (the brain region where induction was observed) and examined the effects of such knockdown on LTM of context fear conditioning. Animals were implanted with bilateral cannulae directed to the amygdala. Cannulae placement confirmation using thionine was highly precise and consistent, showing that injectors specifically targeted regions just above the amygdalar complex. Importantly, only the behavioral data of animals for which thionine staining confirmed correct cannulae localization were used to determine the effects of the oligonucleotides treatment. Representative schematics illustrating the distribution of cannulae placements throughout the amygdala for animals used in our antisense behavioral experiments are depicted in Figure 3(a). Additionally, we examined the diffusion and incorporation of* RAG1* antisense oligonucleotides in the amygdala (Figures 3(b) and 3(c)) through consecutive rostrocaudal sections. Diffusion of the microinfused FITC-*RAG1* antisense oligonucleotide was concentrated within the anterior, posterior, and ventral basolateral amygdala (BLA). It was also observed that FITC-*RAG1* antisense oligonucleotide was clearly incorporated into the cells within these amygdalar regions (Figure 3(d)).

**Figure 4:**
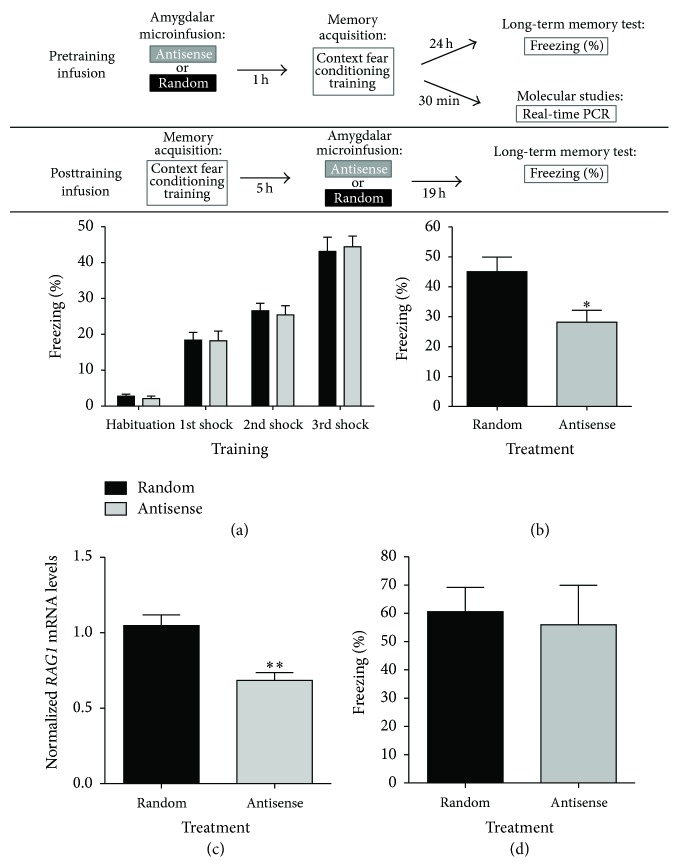
*RAG1* antisense amygdalar treatment impaired consolidation of context fear conditioning. Top panel: diagram depicting the experimental design of these experiments for* pretraining* or* posttraining* amygdalar antisense or random oligonucleotide microinfusion experiments. In the pretraining microinfusion experiments, mice received* RAG1* antisense or random bilateral oligonucleotide microinfusions directed at the amygdala 1 h before conditioning followed by either LTM testing or molecular evaluation. LTM was tested 24 h after conditioning. For molecular evaluation of antisense treatment effectiveness, another group of mice was sacrificed 30 min after conditioning and amygdalar RNA was used for real-time PCR. In the posttraining microinfusion experiments, mice were conditioned, returned to their home cages, and received microinfusions of antisense or random oligonucleotides 5 h after training and returned to their home cages until next day. Nineteen (19) hours later (24 h after conditioning), mice were reexposed to the conditioning chamber without any shocks in order to test LTM. (a) Mice receiving either* RAG1* antisense or random oligonucleotide treatment displayed no significant differences during memory acquisition measured as the progressive enhancement of freezing behavior (Two-Way ANOVA, Treatment Factor: *F*(1,0.8457) = 0.01015, *P* > 0.9; Training Factor *F*(3,7863) = 94.37, ^*∗∗∗*^ *P* < 0.0001; Interaction: *F*(3,7.457) = 0.08950, *P* > 0.9). Bonferroni posttesting analysis did not identify significant differences between the groups during the habituation or the 1st, 2nd, or 3rd trials of training (*P* > 0.05, each comparison), indicating that both groups were similarly capable of learning the task. (b) LTM was tested 24 h after conditioning. The bar graph shows that, unlike the results obtained for acquisition, mice treated with* RAG1* antisense gapmer oligonucleotides displayed significantly less percent freezing to the conditioning context than random oligonucleotide controls during the LTM test (Student's *t*-test; *t*_(25)_ = 2.602; ^*∗*^ *P* < 0.05). (c) The molecular effectiveness of our knockdown by gapmer antisense oligonucleotide of* RAG1* in the amygdala was determined by quantitative real-time PCR. Mice were infused 1 h before context fear conditioning with bilateral* RAG1* antisense or random oligonucleotides and decapitated 30 min after conditioning.* RAG1* mRNA normalized against* gapdh* mRNA showed that treatment with* RAG1* antisense gapmer oligonucleotides effectively knocked down the levels of* RAG1* amygdalar mRNA compared to the random controls (Student's *t*-test; *t*_(16)_ = 3.947; ^*∗∗*^ *P* < 0.005). No significant differences in the levels of* gapdh* were observed between treatments (data not shown). (d) We used 5 h posttraining amygdalar microinfusions of* RAG1* antisense oligonucleotides or random controls with a different set of animals without any pretraining infusion and LTM was tested 24 h after training. Unlike in the pretraining microinfusion experiments, both the antisense and random posttraining-infused mice displayed similar levels of conditioned freezing during the LTM test (Student's *t*-test; *t*_(12)_ = 2.835; *P* > 0.7).

We next evaluated the effects of amygdala* RAG1* antisense treatment on LTM of context fear conditioning. Male C57BL/6 mice were microinfused bilaterally into the amygdala with* RAG1* antisense or random oligonucleotides (*n* = 16, each) 1 h before conditioning training. The top panel of Figure 4 depicts the experimental design of Figures 4(a)–4(c) and 4(d), respectively. We used the 1 h pretraining infusion time point in order to allow for the antisense oligonucleotides to be diffused into the brain parenchyma and be taken up by neuronal cells prior to subjecting the animals to context fear conditioning. For context fear conditioning, mice were placed inside a conditioning context (the chamber, CS) before receiving three consecutive foot shocks (US). As seen in Figure 4(a), mice receiving either antisense or random acquired the task normally, displaying no significant differences in acquisition of fear conditioning, measured as the progressive enhancement of freezing behavior during a 60 s after-shock period. As stated, Two-Way RM ANOVA followed by Bonferroni posttesting found no effect by treatment, although animals in both groups acquired the task, demonstrating that the infusions did not impair the animals' response in developing and expressing fear during the conditioning experience (Treatment Factor: *F*(1,0.8457) = 0.007142, *P* > 0.9; Training Factor *F*(3,7863) = 109.8, ^*∗∗∗*^ *P* < 0.0001; Interaction: *F*(3,7.457) = 0.1041, *P* > 0.9; and Subject Matching: *F*(25,118.4) = 1.653, ^*∗*^ *P* < 0.05). Posttesting analysis did not identify any specific significant differences between the groups during the habituation or the 1st, 2nd, or 3rd trials of training (*P* > 0.05, each comparison). These results indicate that both groups were similarly capable of learning the task. LTM was then tested 24 h after conditioning by placing animals back into the conditioning chamber. During the LTM test, mice remained in the chamber for 4 min in order to measure their freezing response to the context (CS). The bar graph in Figure 4(b) shows that during LTM testing mice treated with* RAG1* antisense gapmer oligonucleotides displayed significantly less percent freezing to the conditioning context than random oligonucleotide controls (Student's *t*-test; *t*_(25)_ = 2.602; ^*∗*^ *P* < 0.05). Thus, pretraining antisense microinfusion into the amygdala significantly impaired LTM as tested 24 h after conditioning.

To confirm the molecular effectiveness of our knockdown by gapmer antisense oligonucleotides of* RAG1* in the amygdala, we performed quantitative real-time PCR experiments. The antisense approach is a well-established technique and has been extensively used in the brain to assess memory function [50–54]. Our* RAG1* antisense gapmer targets the translation initiation codon, thus causing a knockdown of RAG1 protein by translational repression [55–58]. Additionally, the gapmer oligonucleotide contains a central block of deoxynucleotides sufficient to induce the endogenous mechanism of RNA:DNA duplex degradation by ribonuclease H (RNase H) cleavage and thus targeted degradation of mRNA hybridized with the antisense oligonucleotide. Moreover, the antisense gapmer is flanked by blocks of 2′-*O*-methyl modified ribonucleotides that protect the internal block from exonuclease degradation [58–61], which increases the stability and half-life of the unhybridized gapmer oligonucleotide itself. Testing the effectiveness of antisense treatment in the brain has been used by us and others by measuring knockdown of target gene mRNA levels using real-time PCR [62–64]. To test the molecular effectiveness of the* RAG1* gapmer antisense oligonucleotide, mice were infused 1 h before context fear conditioning with bilateral* RAG1* antisense (*n* = 8) or random (*n* = 10) oligonucleotides. Amygdalar* RAG1* mRNA expression was analyzed in trained mice sacrificed 30 min after conditioning, the time point of highest expression seen in the time course studies (see Figure 1(b)).* RAG1* mRNA was normalized against* gapdh* mRNA as above. As seen in Figure 4(c), treatment with* RAG1* antisense gapmer oligonucleotides effectively knocked down the levels of* RAG1* amygdalar mRNA compared to the random controls (Student's *t*-test; *t*_(16)_ = 3.947; ^*∗∗*^ *P* < 0.005). No significant differences in the levels of* gapdh* were observed between treatments (data not shown). These results show the effectiveness and selectivity of the antisense treatment on knocking down* RAG1* expression.

The results presented so far suggest that RAG1 is required in the early phase of LTM consolidation. Molecular events leading to LTM consolidation occur within the early time window during the first 6 h after training, although consolidation processes may last from hours to days and even the transfer of memories to other cortical regions might take longer time periods [65–67]. For instance, it is reported that, in rodents, fear memories require distinct molecular and temporal transcriptional/translational events lasting up to 6 h [65, 66, 68, 69] after learning experiences. More related to this work, our previously reported findings suggested that DNA ligase-dependent NHEJ events, which are associated in general with the repair of DNA DSBs, but also with V(D)J recombination processes in the immune system [26, 70, 71], are also induced rapidly in the hippocampus after context fear conditioning [32]. In addition, we also reported that the function of the flap structure-specific DNA endonuclease 1 (Fen1), known to be involved in DNA recombination/repair processes [29, 72], is induced in the amygdala 3 h after conditioned taste aversion (CTA) learning and is required for LTM consolidation [29]. Hence, for our next set of experiments, we used delayed posttraining amygdalar infusions of* RAG1* antisense oligonucleotides or random controls in order to better assess whether RAG1 is specifically involved in the early stages of consolidation. Animals assigned to the antisense or the random treatments were implanted with cannulae as the animals in the pretraining infusion experiment (Figures 4(a) and 4(b)). Mice of both groups were subjected to context fear conditioning as above, except that these animals had not received pretraining infusions of gapmer antisense or random oligonucleotides before exposing them to the task. Immediately after training, mice were returned to their home cages and then subjected to bilateral intra-amygdalar infusions of either* RAG1* antisense or random oligonucleotides 5 h after training. LTM was tested 24 h after conditioning. The results depicted in Figure 4(d) show that, unlike in the pretraining microinfusion experiments (see Figure 4(b)), both the antisense and random posttraining-infused mice (*n* = 7, each group) displayed similar levels of conditioned freezing during the LTM test (Student's *t*-test; *t*_(12)_ = 2.835; *P* > 0.7). Overall, these behavioral results suggest that, for LTM of context fear conditioning to be established, RAG1 is required at early, rather than later, time points following the time of learning experiences.

**Figure 5:**
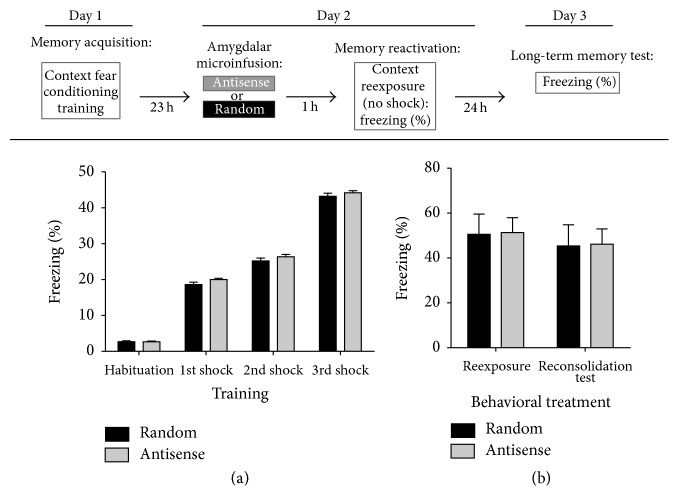
*RAG1* antisense amygdalar treatment does not interfere with reconsolidation of context fear conditioning. To test the effects of* RAG1* gapmer antisense treatment on memory reconsolidation of context fear conditioning, another set of animals was bilaterally implanted with cannulas to target the amygdala. Top panel: diagram depicting the experimental design. On day 1, mice were trained in context fear conditioning and immediately returned to their home cages. Antisense or random oligonucleotides were microinfused into the amygdala 1 h before memory reactivation on day 2. The effect of antisense or random oligonucleotide treatment on LTM reconsolidation was assessed on day 3, 48 h after conditioning. (a) On day 1, mice were microinfused with saline 1 h before training and returned to their home cages immediately after conditioning. Two-Way RM ANOVA and Bonferroni posttesting demonstrated that the infusions did not impair the animals' response in developing and expressing fear during the conditioning experience (Treatment Assignment Factor: *F*(1,8.194) = 3.979, *P* > 0.05; Training Factor *F*(3,3134) = 1725, ^*∗∗∗*^ *P* < 0.0001; and Interaction: *F*(3,1.069) = 0.5881, *P* > 0.6). (b) On day 2, animals were microinfused with either random or antisense gapmer oligonucleotides 1 h prior to a 90 s reexposure period to the conditioning chamber in order to induce memory retrieval and returned to their home cages. For the reconsolidation test, on day 3 (48 h after training), mice were reexposed to the conditioning chamber (CS) for 2 min to measure freezing responses. No significant differences between the freezing responses of antisense or random gapmer oligonucleotide treated animals on day 2 (b) or on day 3 (b) were observed (Two-Way ANOVA: Treatment Factor: *F*(1,3.068) = 0.009017, *P* > 0.9; Training Factor *F*(1,146.5) = 0.4307, *P* > 0.5; Interaction: *F*(1,0.001515) = 0.00004453, *P* > 0.9). Bonferroni posttesting identified no difference between treatments in the reexposure and reconsolidation tests, respectively (*P* > 0.05, each comparison).

Finally, in additional control experiments we tested the effects of* RAG1* gapmer antisense treatment on memory reconsolidation of context fear conditioning. We used this additional control because our previous studies using the DNA ligase inhibitor ara-C, which blocks DNA repair by blocking NHEJ activity [31, 73, 74], showed that treatment with this inhibitor blocked LTM consolidation, but not reconsolidation, of context fear conditioning [32]. Such results also suggested that DNA recombination/repair mechanisms are specific to the initial stages of LTM consolidation and are not involved in memory reconsolidation processes activated after the retrieval of memories that have already been established. For these experiments (see the top panel of Figure 5 for depiction of the experimental design), a set of animals was bilaterally implanted with cannulas to target the amygdala (see Section 2). This protocol was performed similar to [32, 38]. On day 1, mice were microinfused with saline (as a stress control) and were trained 1 h later in context fear conditioning. All animals were returned to their home cages immediately after training. On day 2 (24 h after training), animals were microinfused with either random (*n* = 5) or antisense (*n* = 6) gapmer oligonucleotides. Microinfusions were given 1 h prior to a 90 s reexposure period to the conditioning chamber in order to induce memory retrieval. Next, all animals were returned to their home cages. Finally, on day 3 (48 h after training), all mice were again exposed to the conditioning chamber (CS) for 2 min to measure freezing responses. As seen in Figure 5(a), no significant differences were observed during acquisition between the animals assigned to antisense or random* RAG1* gapmer oligonucleotide treatment. Accordingly, Two-Way RM ANOVA followed by Bonferroni posttesting found no effect by treatment assignment, although animals in both groups acquired the task normally, demonstrating that the infusions did not impair the animals' response in developing and expressing fear during the conditioning experience (Treatment Factor: *F*(1,8.194) = 3.979, *P* > 0.05; Training Factor *F*(3,3134) = 1725, ^*∗∗∗*^ *P* < 0.0001; and Interaction: *F*(3,1.069) = 0.5881, *P* > 0.6). Posttesting analysis did not identify any specific significant differences between the groups during the habituation or the 1st, 2nd, or 3rd trials of training (*P* > 0.05, each comparison). These results indicate that both groups were similarly capable of learning the task. More importantly, as seen in Figure 5(b), with respect to our question of whether amygdalar* RAG1* antisense treatment impaired memory reconsolidation or not, the results identified no significant differences between the freezing responses of antisense or random gapmer oligonucleotide treated animals on day 2 (memory retrieval/reexposure test) or on day 3 (reconsolidation test) (Two-Way ANOVA: Treatment Factor: *F*(1,3.068) = 0.009017, *P* > 0.9; Training Factor *F*(1,146.5) = 0.4307, *P* > 0.5; Interaction: *F*(1,0.001515) = 0.00004453, *P* > 0.9). Bonferroni posttesting identified no difference between treatments in the reexposure and reconsolidation tests, respectively (*P* > 0.05, each comparison). Overall, these results are congruent with our previous findings suggesting that DNA DSB repair mechanisms are possibly relevant only for the initial stages of LTM consolidation and are not utilized when established memories are retrieved or reactivated [32].

## 4. Discussion

We previously reported that a DNA repair system involving DNA ligase function and NHEJ activity is induced and required for memory consolidation [32]. Additionally, studies with TdT, a specialized polymerase involved in V(D)J recombination [71, 75, 76], showed that TdT expression is induced as a result of new experience in brain regions involved in memory formation and is required for normal learning and memory in mice [30]. Our subsequent studies identified the endonuclease Fen1, known to be involved in DNA recombination/repair processes, as being induced after associative learning and being required for LTM formation [29]. Moreover, in more recent studies, mice exposed to a novel environment [27] were shown to accumulate DNA DSBs in the brain, including the hippocampus. These DNA lesions were shown to be transient, as they were repaired after 24 h, highlighting the importance of the DNA repair of such DSBs, which if left unrepaired could ensue in neuronal dysfunction, and also suggesting that they could be related to learning processes associated with exposure to novel environments. In addition, more recent studies showed that DNA DSBs are introduced in the promoters of early-response genes and are required for their induction in response to neuronal activity, synaptic plasticity processes, and context fear conditioning [28]. Accordingly, these studies are reminiscent of our previous findings showing that NHEJ activity repairing DNA DSBs is rapidly induced in the hippocampus, but not the insular cortex, following context fear conditioning in mice [32].

Our present studies further support the notion that DNA recombination/repair machineries particularly involving DNA endonucleases might be involved in learning and memory processes. Specifically, we identified and characterized the expression of* RAG1* in wild type C57BL/6 mice brain during context fear conditioning and confirmed that* RAG1* expression is required in the amygdala for early consolidation of fear memory. These findings further confirm previous reports suggesting the role of* RAG1* in some behavioral paradigms and support the notion that DNA recombination/repair mechanisms may be required in LTM.

### 4.1. *RAG1* Induction in the Amygdala Is Associated with Context Fear Conditioning Learning

Using quantitative real-time PCR, we compared hippocampal and amygdalar* RAG1* expression of context fear conditioning-trained animals during a time course of 15 min, 30 min, and 1 h after conditioning. The levels of hippocampal* RAG1* mRNA after training remained similar to Naïve basal levels. On the other hand, we did observe a rapid and transitory induction of* RAG1* mRNA in the amygdala between 15 and 30 min, which returned to baseline at 1 h, suggesting that* RAG1* is tightly regulated at the level of transcription in the amygdala compared to the hippocampus. This is consistent with previous reports supporting the major role of the amygdala in fear memory, compared to the hippocampus [77–79]. Additionally, we compared amygdalar expression of* RAG1* in conditioned animals and mice subjected to the individual components of this aversive learning paradigm: context-only and shock-only. Interestingly, we found that* RAG1* mRNA levels are significantly higher only in context fear conditioning-trained animals compared with those trained with the individual, unpaired, components of the associative paradigm, as well as with naive. The specific induction of* RAG1* in conditioning paired stimuli suggested that such induction was not merely a correlate of fear itself, but it could be involved in associative learning and LTM formation. In contrast, previous reports have found that immediate early genes involved in general neuronal activation such as* c-fos* are induced in context fear conditioning, but also in context-only (hippocampus) and shock-only (amygdala) animal groups [80–83]. However, the specific induction of* RAG1* to conditioned animals in the amygdala suggests that it does not correspond to the response of a general pattern of neuronal activation.

Because of the widely established role of RAG1 during DNA rearrangement, a process thought to be highly specific to nuclei of immune cells, we wanted to determine the cellular localization of this endonuclease within the amygdalar tissue after context fear conditioning. Thus, after ruling out the possibility that the observed changes in* RAG1* mRNA after conditioning could be due to the presence of residual blood cells in the tissues examined, we performed immunofluorescence analysis of the RAG1 protein. Importantly, for immunofluorescence, brains from trained animals sacrificed 1 h after conditioning were also perfused with PBS1X to remove residual blood and subsequently with paraformaldehyde for tissue fixation. Results demonstrated that NeuN, a marker for neuronal nuclei, was colocalized with RAG1 expression, suggesting that RAG1-positive cells in the amygdala after training are predominantly neurons. Interestingly, not all neurons marked with NeuN were colabeled with RAG1, suggesting that DNA recombination/repair machineries associated with this factor might be restricted to only a subset of cells in the amygdala after context fear conditioning.

### 4.2. *RAG1* Is Required for Consolidation, but Not Reconsolidation, of Context Fear Conditioning

The amygdala plays a critical role for LTM consolidation and for the representation of the US component of context fear conditioning [78, 79, 84–86]. We found that* RAG1* mRNA is specifically induced in the amygdala, but not in the hippocampus, after training. Interestingly, some reports have demonstrated that the effect of amygdalar lesions on fear memory impairment is stronger than the effect of hippocampal lesions, suggesting a major role of the amygdala in fear memory [78, 79]. Hence, we decided to extend our studies by using experiments addressing the functional role of amygdalar* RAG1* on LTM of context fear conditioning. We found that* RAG1* antisense, but not random, oligonucleotides were effective in selectively suppressing the levels of amygdalar* RAG1* mRNA and also impaired LTM of context fear conditioning without affecting acquisition of the task. Animals microinfused with antisense or random oligonucleotides 5 h after conditioning showed no effects in LTM by the antisense targeting, suggesting that* RAG1* is only required in the early phase of LTM formation. Furthermore,* RAG1* antisense or random oligonucleotides infused into the amygdala 1 h prior to memory reactivation of previously trained untreated mice resulted in no effect in either memory retrieval or memory reconsolidation. Consistent with these findings, we previously found that administering ara-C, an inhibitor of DNA ligase and of NHEJ activity shown to block LTM consolidation of context fear conditioning, just prior to memory reactivation had no effect in memory reactivation or memory reconsolidation [32]. Altogether, these results suggest that, unlike CREB inactivation and general protein synthesis inhibition [38, 87, 88], blockade of DNA recombination/repair processes during memory reactivation does not interfere with reconsolidation of fear conditioning [32]. The fact that mechanisms associated with the introduction of DNA DSBs are restricted to the early phases of LTM consolidation and are not activated as a result of memory retrieval might represent a mechanism for a balance that would allow neurons to maintain their integrity by not overriding their mechanisms for DNA repair, which could occur if these lesions were to be introduced every time an established memory is retrieved.

The results presented above are consistent with previous studies using* RAG1*-knockout (*RAG1* ^−/−^) and* RAG1*-deficient mice (RAG1^−/+^).* RAG1* ^−/−^ mice showed reduced levels of fearfulness for some measures of fear-motivated behavior in both the open-field behavior test and the elevated-plus maze [36]. Additionally,* RAG1* ^−/+^ exhibited impaired social recognition memory [37]. Moreover,* RAG1* ^−/−^ mice showed memory impairment compared with wild type in the Morris water maze [89]. These findings with* RAG1* ^−/−^ and* RAG1* ^−/+^ mice are interesting; however, it is known that, in some cases, such gene targeting models might be masked by compensatory mechanisms, or developmental and physiological side effects, sometimes undetectable, because the mutation targets all cells [50, 90–92]. For instance, mice presenting inactivating mutations or deletion of the* RAG1* gene show severe combined immunodeficiency (SCID) caused by small lymphoid organs, impaired development of B and T lymphocytes, and inability to perform V(D)J recombination, but with no obvious neuroanatomical abnormalities [93–97]. In contrast,* RAG1* ^−/+^ mice in which one copy of the* RAG1* gene is deleted and thus which are heterozygous for the* RAG1 gene* (−/+), are immunocompetent and indistinguishable from wild type mice, displaying normal lymphocytes differentiation and V(D)J recombination [93, 95]. Nevertheless, neither of the knockout or heterozygous models mentioned above have regional or temporal control for* RAG1* gene inactivation. The antisense oligonucleotide approach has the advantage of brain region specificity in the region of interest (the amygdala) at a specific time point. For instance, knockout mice targeting the transcription factor encoded by the immediate early gene* c-fos* show normal acquisition and LTM [50]. However, acute knockdown with antisense oligonucleotide to specific brain regions involved in the learning paradigm inhibits LTM of wild type mice [50], suggesting that compensatory mechanisms are activated in the absence of Fos-mediated transcription in knockout models. Conversely, CREB mutants and knockouts display memory disruption in fear conditioning and in a wide range of behavioral paradigms [11, 98, 99]. Similarly, targeted injection of CREB antisense into the hippocampus or into the amygdala results in disruption of LTM in a variety of tasks [53, 100]. Similar to our studies reported here with gapmer antisense oligonucleotides, a study using shRNAs targeting* RAG1* delivered with lentiviral vectors into the CA3 region of the rat hippocampus was used to assess the effects of suppressing* RAG1* expression on spatial learning in the Morris Water maze [101]. These studies demonstrated that suppressing* RAG1* expression in the hippocampal CA3 region does impair spatial learning in rats. The authors also examined the effects of such suppression on context fear conditioning. Similar to our findings here with respect to* RAG1* amygdalar knockdown in mice, their findings suggest that hippocampal expression of* RAG1* is necessary for LTM of context fear conditioning, although no experiments were done to examine whether changes in* RAG1* expression occurred either in the hippocampus or in the amygdala as a result of learning in either of the paradigms. With respect to the findings on context fear conditioning, it is important to state that in our studies reported here we did not observe hippocampal induction of* RAG1* mRNA levels after conditioning (see Figure 1(a)); that is,* RAG1* mRNA levels remained at their basal Naïve levels at each of the time points examined. We cannot rule out, however, and in light of the study mentioned above, that the basal constitutive expression of* RAG1* in the hippocampus after context fear conditioning may play a role in LTM, as well as the induced amygdalar expression. Finally, as suggested previously [102] it is possible that different molecular mechanisms operate in consolidation of distinct learning paradigms, either spatial learning or context fear conditioning, which might involve regulated or constitutive* RAG1* expression and function in distinct regions of the brain.

Our findings on* RAG1* induction and functional knockdown studies with antisense, together with previous reports on* RAG1* ^−/+^ [36, 89] and* RAG1* ^−/+^ [58] mouse models, support the possibility that this DNA endonuclease, which introduces DNA DSBs, is required for associative memory formation. Additional support for the required role of* RAG1* in LTM is provided by the observation that* RAG1* induction is specifically related to the pairing of individual conditioning stimuli (Figure 1(c)) and the fact that the amnesic effect of amygdalar knockdown of RAG1 is evident 24 h after acquisition of context fear conditioning (Figure 4(b)). The crucial question at this time is whether RAG1 indeed is involved in introducing DNA DSBs in response to learning and whether the repair of such DNA lesions is required for LTM. Although the data presented here and that reported previously concerning the experience-dependent expression of factors specifically related to V(D)J recombination in brain regions involved in memory formation further strengthen this view [27–32], additional published data could suggest that such changes may not be associated with the introduction of DNA DSBs and DNA repair. For example, a cytoplasmic form of the P13 kinase member ataxia-telangiectasia mutated (ATM) [103], known to play a key role in DNA DSB repair, was shown to play an important role in synaptic plasticity. However, the studies also suggested that this form of cytoplasmic ATM is not involved in the key role of DNA DSB repair ascribed to the nuclear form of the protein. The authors suggested that cytoplasmic ATM plays cellular roles in neurons that are independent of its role in responding to DNA lesions. Thus, we cannot rule out at this time that our findings with RAG1 may be unrelated to its role in introducing DNA DSBs that is well documented in the immune system. However, in the studies presented here localization of RAG1 after conditioning was concentrated in neuronal nuclei in the amygdala, colocalizing with the neuronal nuclear marker NeuN, suggesting that the role of RAG1 in the amygdala after learning is associated with nuclear machineries such as DNA recombination/repair. Moreover, our previous reports on experience-dependent induction of NHEJ activity and DNA repair machineries suggest that DNA DSBs are generated as a result of learning, perhaps as a consequence of induced endonuclease activity such as RAG1, and subsequent DNA repair by induction of NHEJ/DNA ligase pathway. Overall, based on these findings we propose that DNA recombination/repair pathways [23], involving a V(D)J-like mechanism using* RAG1*, DNA ligase/NHEJ [31–33], Fen1 [29], and TdT [30], possibly operate together with epigenetic and transcriptional/translational regulation for LTM formation. Such a mechanism could be responsible not only for regulating the activity of gene promoters and thereby gene expression, but also for increasing the diversity of the repertoire of genes and proteins required for synaptic plasticity processes and the establishment of specific connectivity of neuronal networks and/or the specific patterns of gene expression involved in the establishment of specific long-term memories.

## 5. Conclusion

The present studies have identified and characterized,* RAG1*, a gene encoding a key endonuclease that introduces DNA DSBs in recombination processes of the immune system, required for LTM formation of aversive experiences. In immune cells, somatic DNA rearrangement is initiated by RAG1, which exerts endonuclease activity upon the RSSs of V(D)J gene segments resulting in enhanced diversity of antigen receptor genes [42, 71, 104, 105]. Because of the well-known molecular function of RAG1 as the DNA endonuclease initiating V(D)J recombination of T-cell and immunoglobulin receptors in lymphoid cells, our findings support the proposed role of DNA recombination/repair mechanisms in LTM processes [23, 27–32]. Introduction of DNA DSBs, DNA repair, and DNA rearrangement are not inconsistent with synaptic plasticity models and mechanisms previously described. Thus, an integrated control of the introduction of DNA DSBs, DNA repair, DNA rearrangement, epigenetics, and transcriptional and translational mechanisms may orchestrate gene regulation in memory formation.

## Supplementary Material

In order to confirm the molecular identity of the PCR products amplified from thymus, hippocampus and amygdala tissues utilizing primers targeting *RAG1*, we carried out several analyses. Supplementary Figures 1A and 1B depict representative amplification (to show the cycle thresholds, Ct) and melting plots, respectively, for *RAG1* and *gapdh* by quantitative real-time PCR. As seen in Supplementary Figure 1B, melting temperature analysis consistently confirmed the formation of one specific amplification product per primer set. In addition, the gapdh amplification product appeared with a higher peak compared to that of the *RAG1* amplification product, as expected based on the results shown in Supplementary Figure 1A. We also carried out standard PCR analysis for *RAG1*. A representative agarose gel depicting the results of such analysis is shown in Supplementary Figure 1C, demonstrating that the *RAG1* primer sets amplified a single PCR product in the three tissues examined. These products were cloned and sequenced following PCR and agarose gel electrophoresis. Supplementary Figure 1D shows the sequence eletropherograms from PCR products amplified from amygdala, hippocampus and thymus tissues. The resulting sequences from each PCR product were subjected to sequence alignment using ClustalW2, comparing them to each other and to *Mus musculusRAG1* reference sequence NM_009019.2 (see Supplementary Figure 1E). Finally, the molecular identity of *RAG1* PCR products from amygdala, hippocampus and thymus was confirmed using mouse genome BLAST analysis, which showed a 100% match identity to Mus musculus *RAG1* (Ref | NM_009019.2) with an E-Value of 2e-19 (see Supplementary Figure 1F).
