# Supplementary material for: Identification and Characterization of the V(D)J Recombination Activating Gene 1 in Long-Term Memory of Context Fear Conditioning
Source: Neural Plast. 2015 Dec 30;2016:1752176. doi: 10.1155/2016/1752176 (PMC4710954; doi:10.1155/2016/1752176)
Supplement: Supplementary file 1 — In order to confirm the molecular identity of the PCR products amplified from thymus, hippocampus and amygdala tissues utilizing primers targeting RAG1, we carried out several analyses. Supplementary Figures 1A and 1B depict representative amplification (to show the cycle thresholds, Ct) and melting plots, respectively, for RAG1 and gapdh by quantitative real-time PCR. As seen in Supplementary Figure 1B, melting temperature analysis consistently confirmed the formation of one specific amplification product per primer set. In addition, the gapdh amplification product appeared with a higher peak compared to that of the RAG1 amplification product, as expected based on the results shown in Supplementary Figure 1A. We also carried out standard PCR analysis for RAG1. A representative agarose gel depicting the results of such analysis is shown in Supplementary Figure 1C, demonstrating that the RAG1 primer sets amplified a single PCR product in the three tissues examined. These products were cloned and sequenced following PCR and agarose gel electrophoresis. Supplementary Figure 1D shows the sequence eletropherograms from PCR products amplified from amygdala, hippocampus and thymus tissues. The resulting sequences from each PCR product were subjected to sequence alignment using ClustalW2, comparing them to each other and to Mus musculusRAG1 reference sequence NM_009019.2 (see Supplementary Figure 1E). Finally, the molecular identity of RAG1 PCR products from amygdala, hippocampus and thymus was confirmed using mouse genome BLAST analysis, which showed a 100% match identity to Mus musculus RAG1 (Ref | NM_009019.2) with an E-Value of 2e-19 (see Supplementary Figure 1F). [file 1752176.f1.pptx]

## Slide 1
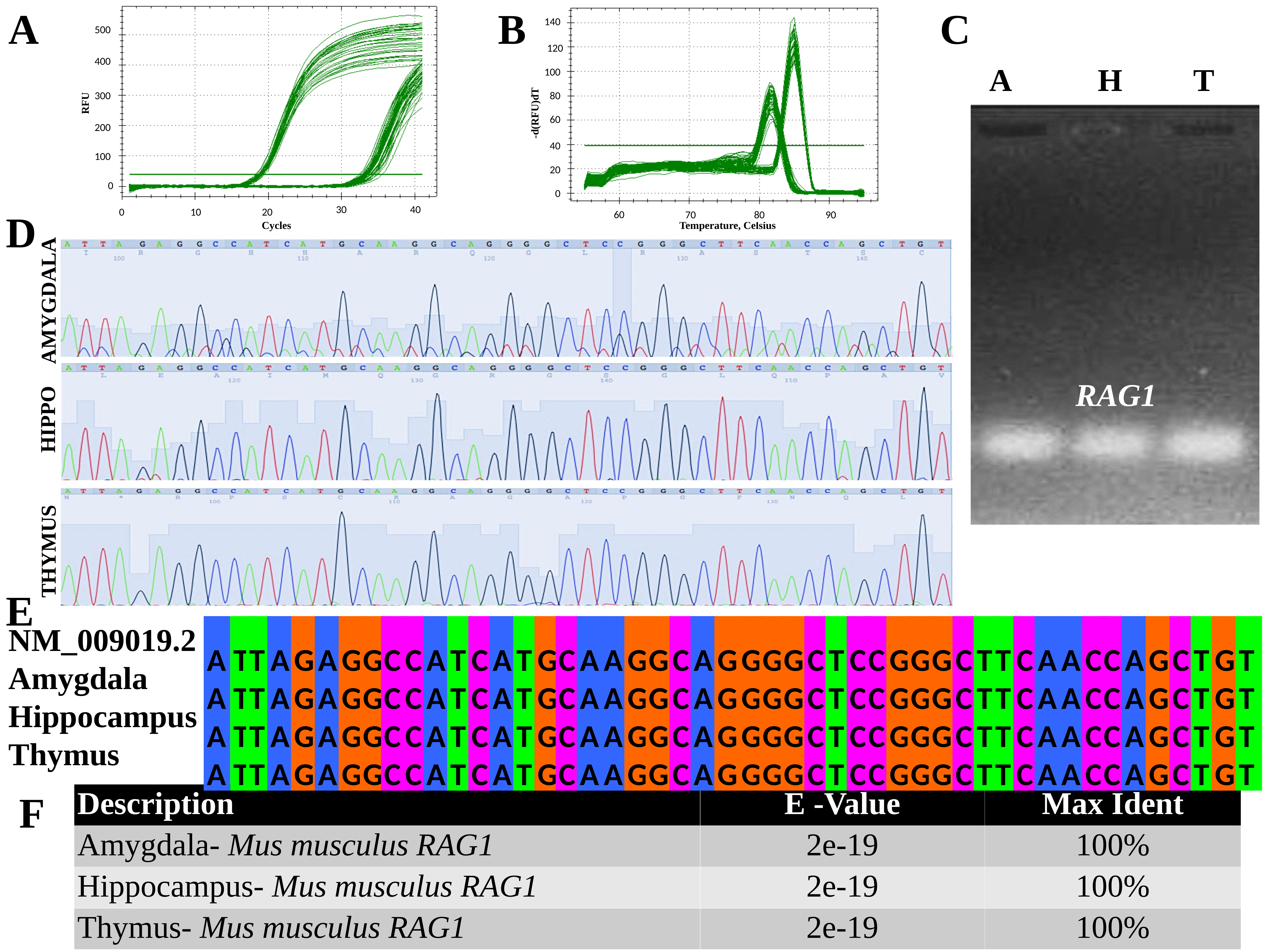

C
A
B
140
500
120
400
A H T
100
300
80
RFU
-d(RFU)dT
60
200
40
100
20
0
0
30
40
0
10
D
20
60
70
80
90
Cycles
Temperature, Celsius
AMYGDALA
RAG1
HIPPO
THYMUS
E
A
A
A
A
T
T
T
T
T
T
T
T
A
A
A
A
G
G
G
G
A
A
A
A
G
G
G
G
G
G
G
G
C
C
C
C
C
C
C
C
A
A
A
A
T
T
T
T
C
C
C
C
A
A
A
A
T
T
T
T
G
G
G
G
C
C
C
C
A
A
A
A
A
A
A
A
G
G
G
G
G
G
G
G
C
C
C
C
A
A
A
A
G
G
G
G
G
G
G
G
G
G
G
G
G
G
G
G
C
C
C
C
T
T
T
T
C
C
C
C
C
C
C
C
G
G
G
G
G
G
G
G
G
G
G
G
C
C
C
C
T
T
T
T
T
T
T
T
C
C
C
C
A
A
A
A
A
A
A
A
C
C
C
C
C
C
C
C
A
A
A
A
G
G
G
G
C
C
C
C
T
T
T
T
G
G
G
G
T
T
T
T
NM_009019.2
Amygdala
Hippocampus
Thymus
F
| Description | E -Value | Max Ident |
| --- | --- | --- |
| Amygdala- Mus musculus RAG1 | 2e-19 | 100% |
| Hippocampus- Mus musculus RAG1 | 2e-19 | 100% |
| Thymus- Mus musculus RAG1 | 2e-19 | 100% |
